# Factors disturbing undergraduate students’ interaction during lectures: A university-based survey

**DOI:** 10.12669/pjms.38.7.5101

**Published:** 2022

**Authors:** Muhammad Imran, Mukhtiar Baig, Manal Abdulaziz Murad, Saleh H Almurashi

**Affiliations:** 1 Muhammad Imran, FCPS, FRCS, MCPS-HPE. Department of Surgery and Medical Education Unit, Faculty of Medicine, Rabigh, King Abdulaziz University, Jeddah, Saudi Arabia; 2 Mukhtiar Baig, PhD, MHPE. Department of Clinical Biochemistry and Medical Education Unit, Faculty of Medicine, Rabigh, King Abdulaziz University, Jeddah, Saudi Arabia; 3 Manal Abdulaziz Murad, MBBS, ABFM, JBFM, Department of Family Medicine, Faculty of Medicine, King Abdulaziz University Hospital, Jeddah, Saudi Arabia; 4 Saleh H. Almurashi, MBBS. Intern in the Department of Medicine, King Abdulaziz University Hospital, Jeddah, Saudi Arabia

**Keywords:** Active participation, Education, Factors Influencing, Lecture, Undergraduate

## Abstract

**Objectives:**

To explore the factors that disturb students’ interaction during lectures and interfere with their active participation.

**Methods:**

This cross-sectional study was conducted at King Abdulaziz University (KAU), Jeddah, Saudi Arabia. The study lasted approximately nine months, beginning on May 9, 2018, and ending on February 6, 2019. Students of different faculties participated in the study. A valid questionnaire was used after piloting. Four major categories were defined: classmate factors (CMFs), teacher-related factors (TRFs), personal factors (PFs), and class environment factors (CEFs).

**Results:**

A total of 658 students were included. Among all, 428 (65%) were females, and 230 (35%) were males. The comparison of main categories showed that CMFs, TRFs, PFs, and CEFs disturbed students’ interaction 74%, 55%, 50%, and 84%, respectively. The comparison of the factors disturbing students’ interaction showed that females were more disturbed by the CMFs (p=0.036) and CEFs (p<0.001) than males. CMFs, PFs, and CEFs disturbed more married students’ than unmarried. CMF more disturbed science faculty students’ interaction compared to all other groups. CEF showed less disturbance among Engineering/Math group students’ interaction compared to other groups. The male gender and sixth-year students were the predictors of TRF disturbance, while the married students were the predictors of disturbance by PF.

**Conclusion:**

Several factors (PF, CMF, TRF, and CEF) disturbed students’ interaction during a lecture. Additionally, the male gender, married students, and sixth-year students were the associated factors of disturbed interaction during a lecture. We suggest that teachers and educational leaders need to devise a policy to overcome these factors to provide a conducive learning environment.

## INTRODUCTION

Lectures are being used worldwide and are considered a necessary instructional tool in undergraduate medical education, and students still perceive it as a good strategy. Usually, the purpose of the lecture is to inspire the students to learn, while several students consider it a method for preparing for assessment. However, traditional lectures have several associated intrinsic disadvantages, such as waned attention and declined comprehension of students.^1^ Conversely, lectures can be interactive with the active participation of students.^2^ It is evident from the literature that students’ active participation enhances their learning process.^3^

Different factors can influence a student to participate during a session, for instance, environmental factors such as classroom size and seat-arrangement, personal traits of students, the role of facilitator, and peer-factors.^4^ Even factors, such as light, seating arrangement, audio-visuals, room temperature, comfort, and technical aspects, can affect learning. Students’ active participation during a face-to-face class can be attributed to certain factors. The instructor’s role carries paramount importance, as certain behaviours of an instructor, such as lack of eye contact, offending behaviour, or speaking too fast, can negatively influence students’ active participation. Similarly, the conduct of peers can affect students to engage in discussions actively.^5^

So, if the factors that interfere with the active participation of students are modified or controlled, this can foster their learning.^6^ A thorough literature search indicated that exactly no similar study is available locally and internationally. However, few studies having a few similar questions are available. Those studies have reported the impact of different factors, which can influence students’ participation.^4^,^5^ Nevertheless, there is a possibility that with the passage of time and technological advancement, the types and influence of factors may change.

Additionally, our study included students of different faculties because it was a university-based survey. Moreover, Rabigh campus is a relatively newly established faculty about 150 km from Jeddah’s main campus.^7^ So, there is a dire need to identify such factors that decrease students` interaction during lectures. We aimed to identify the factors that could disturb undergraduate students’ interaction during a lecture at King Abdulaziz University (KAU), Jeddah, KSA. Identifying these factors would help the stakeholders bring the required changes in the educational environment to facilitate student learning.

## METHODS

This cross-sectional, questionnaire-based study was conducted at KAU, Jeddah, and Rabigh Campuses. Ethical approval from the institution’s research committee was obtained (Approval No. FMR-04-39-H). The study lasted approximately nine months, beginning on May 9, 2018, and ending on February 6, 2019. The confidentiality of participants was maintained, and their names were not disclosed. Students of medicine, engineering, science, mathematics, applied medical sciences, and chemical engineering faculties were invited to participate in the study. The participants were selected by convenience sampling method. The questionnaire - English version with Arabic translation was sent via Google Forms to one thousand students, including male and female students, of different faculties. It was mentioned that filling the questionnaire would be considered their consent for their participation in the study. The students were divided into four groups, for the ease of analysis, according to their faculties. The faculties of medicine and science were included as separate groups, while the faculties of engineering and mathematics were included as a single unit. Students of all other faculties were included in the group named as others.

In the present study, only students of KAU were included, while we excluded the incomplete questionnaires. The sample size was calculated using the Raosoft sample size calculator by taking the population of KAU students 60000, the confidence level of 95%, and the margin of error at 5%. The calculated sample size was 382; however, we sent the questionnaire to 1000 students and included all those students who returned the filled questionnaire. The sample size inflated due to higher attrition in an online questionnaire.

A structured questionnaire was developed that consisted of different questions comprising various factors that might influence a student’s participation during a lecture. Several questions were derived from the previously published studies after modifying a few questions according to our local context.^5^,^8^-^10^ The questionnaire was examined for construct and content validity by two senior faculty members and a medical educator. The questionnaire was validated and reviewed for comprehension on a group of 30 students. After receiving input from students, discrepancies were corrected, and long sentences were rephrased in order to make them simpler, more precise, and unequivocal.

The questionnaire comprised two sections; the first section included demographic questions and the second section comprised 40 questions about the factors that could influence students’ participation during a lecture. Nine questions were related to classmate factors, fifteen questions were related to teacher factors, eleven questions were related to personal factors, and five questions were associated with class environment factors. A five-point Likert scale (from 0-4) was used for each question; strongly agree (4), agree (3), uncertain (2), disagree (1), and strongly disagree (0).

### Statistical analysis

The questionnaire data were stored and analysed on SPSS version 23. The frequencies and percentages were computed. An independent sample t-test and One-way ANOVA was used Binary logistic regression was employed and to identify the predictors of causing a disturbance during class lectures. P-value < 0.05 was considered statistically significant.

## RESULTS

**Fig.1:**
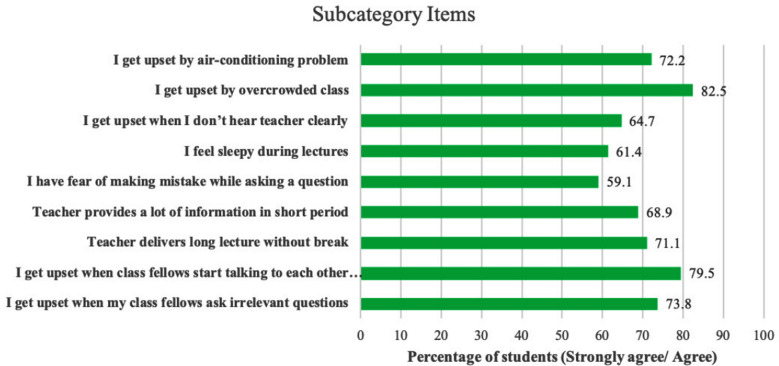
The most common factors disturbing students’ interaction during lecture.

A total of 680/1000 (68%) students filled the questionnaire, while twenty-two questionnaires were not included in the study due to different reasons. Most students, 286(43.5%), were from the medicine faculty. Demographic findings are given in Table-I. The comparison of main categories showed that CMFs, TRFs, PFs, and CEFs disturbed students’ interaction 74%, 55%, 50%, and 84%, respectively (Fig.1).

**Table-I:** Basic characteristics of the participants (N=658)

Variables	n (%)
***Gender***	
Female	428 (65%)
Male	230 (35%)
***Marital status***	
Married	69 (10.5%)
Unmarried	589 (89.5%)
***GPA***	
<=3	101(15.3)
>3	557(84.7)
***Academic year***	
First year	24 (3.6%)
Second year	123 (18.7%)
Third year	161 (24.5%)
Fourth year	190 (28.9%)
Fifth year	89 (13.5%)
Sixth year	71 (10.8%)
***Faculties***	
Medicine	286 (43.5%)
Engineering/Mathematics	137 (20.8%)
Science	135 (20.5%)
others	100 (15.2%)

The comparison of the factors disturbing students’ interaction showed that females were more disturbed by the CMFs (p=0.036) and CEFs (p<0.001), compared to males. CMFs, PFs, and CEFs disturbed more married students’ than unmarried. The post hoc analysis showed that the CMFs more disturbed science faculty students’ interaction than engineering/math, science, and other faculties (p=001, p=<0.001, p=0.006, respectively). PFs also disturbed science faculty students’ interaction than Engineering (p=0.033) and other faculties (p=0.001). Academic-year-wise comparison showed no significant difference in factors disturbing students’ interaction (Table-II).

**Table-II:** Comparison of the factors disturbing students’ interaction during lectures according to different variables.

Parameters	Classmate factors	Teacher-related factors	Personal factors	Class environment factors
***Gender***				
Male n=230	21.53 ±6.13	33.06±12.22	21.99±7.39	13.14±4.29
Female n=428	22.69±7.05	32.70±13.36	23.16±8.07	15.02±3.97
P-value	0.036	0.736	0.069	<.001
***Marital status***				
Unmarried n=589	21.97±6.60	32.52±12.96	22.40±7.56	14.21±4.16
Married n=69	24.92±7.47	35.43±12.74	25.69±9.53	15.63±4.06
P-value	.001	.07	.007	.007
***GPA***				
<=3	21.67±7.64	33.86±13.66	24.08±9.11	14.08±4.42
>3	22.39±6.58	32.64±12.83	22.50±7.58	14.41±4.13
P-value	.32	.38	.06	.47
***Faculty***				
Medical^a^ (n= 286)	21.91±5.59	33.34±13.04	22.58±6.85	14.74±3.51
Engineering/ Mathematics^b^(n= 137)	21.02±6.23	32.25±12.75	22.12±7.19	12.78±4.47
Science^c^(n= 135)	24.80±8.47	33.01±12.49	25.00±9.88	15.20±4.42
Others^d^ (n= 100)	21.68±7.10	31.88±13.75	21.04±7.74	14.29±4.63
P-value	<.001	0.432	0.001,	<.001
***Academic years***				
First Year (n= 24)	21.25±6.19	31.04±10.72	24.29±6.71	14.08±4.28
Second year (n=123)	22.20±6.60	32.83±11.78	23.66±8.05	14.73±3.86
Third year (n= 161)	22.42±7.03	32.53±13.22	22.00±8.09	14.26±4.40
Fourth year (n= 190)	22.61±7.13	32.15±13.15	22.61±8.10	14.71±4.12
Fifth year (n=89)	22.49±6.05	33.85±14.48	22.94±7.36	13.99±4.06
Sixth Year (n= 71)	21.35±6.50	34.65±12.65	22.51±7.24	13.61±4.06
P-value	0.774	0.698	0.519	0.36

The logistic regression analysis showed that TRF’s odds of disturbance were 1.64 times higher among male students than females and 3.16 times higher in sixth-year students than in other year students. PF’s odds of disturbance were 1.82 times higher among married students than unmarried students (Table-III).

**Table-III:** Association of classmate, teacher-related, personal, and class environment factors (Binary logistic regression analysis)

Variables	Classmate factors			Teacher-related factors			Personal factors			Class environment factors		

	B	P-value	OR	B	P-value	OR	B	P-value	OR	B	P-value	OR
Age >22 years	-.299	.208	.742	.165	.424	1.179	.086	.678	1.089	-.170	.543	.843
Male gender	.032	.901	1.032	.497	.028	1.645	.238	.287	1.269	-.588	.050	.555
GPA >3	.387	.111	1.473	-.312	.178	.732	-.244	.285	.784	.521	.068	1.684
Married	.272	.401	1.313	.293	.297	1.341	.602	.033	1.827	.106	.790	1.112
Maths	-.234	.417	.792	-.066	.800	.937	-.358	.162	.699	-.532	.102	.587
Science	.101	.707	1.106	.308	.194	1.361	.280	.239	1.323	-.093	.786	.911
Other	-.147	.574	.863	-.103	.663	.902	-.444	.060	.642	-.407	.203	.666
Second year	.047	.924	1.049	.577	.207	1.782	-.347	.449	.707	.295	.605	1.343
Third year	-.147	.765	.863	.313	.488	1.368	-.711	.118	.491	.287	.609	1.333
Fourth year	.104	.834	1.109	.230	.611	1.259	-.418	.358	.659	.910	.113	2.484
Fifth year	.218	.691	1.243	.472	.337	1.604	-.020	.968	.981	.432	.484	1.541
Sixth year	-.219	.699	.804	1.152	.028	3.164	-.202	.697	.817	.221	.732	1.247
Constant	.918	.102	2.504	-.309	.548	.734	.525	.310	1.691	1.309	.044	3.701

OR= Odds ratio, B= Coefficient for the constant.

## DISCUSSION

Our results showed that students felt disturbed with all the factors, to some extent, mentioned in the questionnaire. The class environment, classmate, and teacher-related factors significantly displayed disturbed interaction. Most of the students’ disturbed by environmental factors such as overcrowded classrooms, air-conditioning issues, and audio-visual problems. The classroom environment concept is not new, and a study mentioned that upgrading the classroom is required for the better learning of students.^11^ A comfortable environment with a good seating arrangement is beneficial for students’ learning and satisfaction.^12^ Overcrowded classrooms can have a negative impact on students’ academic achievements, and in such classes, discipline is another issue. Learners are less motivated, and individual attention and support are lacking.^13^ The educational environment plays an important role in paying concentration during lectures and facilitate a conducive environment for students’ participation in classroom activities.

Regarding instructors’ issue, the majority of the students showed dissatisfaction with too much information in a lecture and long lectures without a break.^14^ It has been evaluated earlier that interaction and break during a lecture have a better impact on students’ learning.^15^ There are issues of students’ active participation in a crowded classroom; however, teachers can handle this problem with well-planned interactive activities.^16^

We suggest that teachers should organize and rehearse their lectures before time. More content in terms of slides compel the teachers to go fast without a break and without involving students with the content. It makes a lecture at a fast pace, lengthy and tedious too.

Interestingly, while giving a response to a question, more than half of the students (60%) responded that ’I feel sleepy during lectures,’ and it raises the question of instructional strategy. However, other possible factors involved, including hot weather, long day schedule, long duration of the lecture, heavy curriculum, dim light, etc., can’t be ignored. So, it is better to design such educational strategies as flipping classrooms, dividing students into small groups, etc. Studies have pointed out issues with traditional lectures, emphasizing engaging activities for the learners.^17^ Mann & Robinson,^18^ in their study, discuss different factors which cause boredom of students in a classroom, and the way a lecture is delivered is a major factor. It is argued that students like lectures if there is good interaction and relevance.^19^ It is reported that active learning strategy, such as flipping the class, is a better learning tool for many subjects^20^, and students appreciate active learning in their learning process.^21^

An additional factor on which many participants exhibit the agreement is the fear of asking questions. It has been advocated that developing communication skills in students should be a part of an instructional strategy.^22^ Mastering these skills is a mandatory competency of a medical student.^23^ So, it is necessary for instructors and educational leaders to develop strategies for excellent communication skills for learners. The blended learning strategy can help the students to overcome their shyness in asking questions in the class.^24^

Another critical issue, highlighted by the students, is classmate-factors. These findings are in accordance with another study.^25^ It is evaluated that peers can be a source of distraction for a learner. In this study, it is argued that an instructor must manage a class.^26^

Students of the faculty of science showed higher disturbed interaction as compared to other faculties. This, again, needs further exploration of the specific issues. It could be due to the concentration required in understanding scientific concepts and mechanisms. Our results did not show any statistical difference among students of different academic years for most factors. However, sixth-year students showed a higher level of disturbed interaction regarding TRF. Another study also reported that senior students have a higher rate of disturbance than their junior counterparts.^25^

It is suggested that teachers should focus more on blended teaching and learning activities, which will lessen the burden on lectures. All factors disturbing the active participation of undergraduate students during lectures will also be avoided.

### Limitations of the study

The first limitation is that this is a survey-based study, so it is prone to response bias. Second, this is a single-centre study, so interaction disturbing factors might be different in other colleges.

## CONCLUSION

Our results showed that PF, CMF, TRF, and CEF disturbed students’ interaction during a lecture. The male gender, married students, and sixth-year students were the associated factors of disturbed interaction during a lecture. We suggest that teachers and educational leaders need to devise a policy to overcome these factors to provide a conducive learning environment.

### Authors’ Contribution:

**MI:** Conceptualized, methodology, project administration, writing draft and responsible and accountable for the work’s accuracy or integrity.

**MB:** Data curation, formal analysis, writing, review and editing.

**MAM, SHM:** Investigation, resources, data collection, review and editing.

## References

[1] Steinert Y, Snell YS (1999). Interactive lecturing:strategies for increasing participation in large group presentations. Med Teach.

[2] Zinski A, Blackwell KT, Belue FM, Brooks WS (2017). Is lecture dead?A preliminary study of medical students'evaluation of teaching methods in the preclinical curriculum. Int J Med Educ.

[3] Hyun J, Ediger R, Lee D (2017). Students'Satisfaction on Their Learning Process in Active Learning and Traditional Classrooms. Int J Teach Learn High Educ.

[4] Abdullah MY, Bakar NR, Mahbob MH (2012). Student's Participation in Classroom:What Motivates them to Speak up?. Procedia Soc Behav Sci.

[5] Yang Z, Becerik-Gerber B, Mino L (2013). A study on student perceptions of higher education classrooms:Impact of classroom attributes on student satisfaction and performance. Build Environ.

[6] Bartholomew KJ, Ntoumanis N, Mouratidis A, Katartzi E, Thogersen-Ntoumani C, Vlachopoulos S (2018). Beware of your teaching style:A school-year long investigation of controlling teaching and student motivational experiences. Learn Instr.

[7] Imran M, Shamim MS, Baig M, Farouq M, Gazzaz ZJ, Al-Mutairi OM (2016). Tale of two cities:comparison of educational environment of two colleges (Jeddah and Rabigh) affiliated with one university. J Pak Med Assoc.

[8] Loftin C, Davis LA, Hartin V (2010). Classroom participation:A student perspective. Teach Learn Nurs.

[9] Mustapha SM, Rahman NS, Yunus MM (2010). Factors influencing classroom participation:a case study of Malaysian undergraduate students. Procedia Soc Behav Sci.

[10] Miller CJ, McNear J, Metz MJ (2013). A comparison of traditional and engaging lecture methods in a large, professional level course. Adv Physiol Edu.

[11] Conway K (2000). Master Classrooms:Classroom Design with Technology in Mind. Resources in Education.

[12] Hill MC, Epps KK (2010). The impact of physical classroom environment on student satisfaction and student evaluation of teaching in the university environment. Acad Educ Leadership J.

[13] Marais P (2016). “We can't believe what we see”:Overcrowded classrooms through the eyes of student teachers. S Afr J Educ.

[14] Mustafa HM, Al-Hamadi A (2017). An Overview on Classrooms'Academic Performance Considering:Non-Properly Prepared Instructors, Noisy Learning Environment, and Overcrowded Classes (Neural Networks'Approach). Brain.

[15] Richardson D (2008). Don't dump the didactic lecture;fix it. Adv Physiol Edu.

[16] Ayu M (2019). Interactive activities for effective learning in overcrowded classrooms.

[17] Wolff M, Wagner MJ, Poznanski S, Schiller J, Santen S (2015). Not another boring lecture:engaging learners with active learning techniques. J Emerg Med.

[18] Mann S, Robinson A (2009). Boredom in the lecture theatre:An investigation into the contributors, moderators and outcomes of boredom amongst university students. Br Educ Res J.

[19] Buchanan T, Palmer E (2017). Student Perceptions of the History Lecture:Does this Delivery Mode have a Future in the Humanities?. J Univ Teach Learn Pract.

[20] Cheng L, Ritzhaupt AD, Antonenko P (2019). Effects of the flipped classroom instructional strategy on students'learning outcomes:A meta-analysis. Educ Technol Res Dev.

[21] Imran M (2019). Analysis of learning and teaching strategies in Surgery Module:A mixed methods study. J Pak Med Assoc.

[22] LeFebvre L, LeFebvre LE, Allen M (2018). Training the butterflies to fly in formation:cataloguing student fears about public speaking. Commun Educ.

[23] Sanson-Fisher R, Hobden B, Waller A, Dodd N, Boyd L (2018). Methodological quality of teaching communication skills to undergraduate medical students:a mapping review. BMC Med Educ.

[24] Baig M, Gazzaz ZJ, Farouq M (2020). Blended Learning:The impact of blackboard formative assessment on the final marks and students'perception of its effectiveness. Pak J Med Sci.

[25] Attia NA, Baig L, Marzouk YI, Khan A (2017). The potential effect of technology and distractions on undergraduate students'concentration. Pak J Med Sci.

[26] Frisby BN, Sexton BT, Buckner MM, Beck AC, Kaufmann R (2018). Peers and Instructors as Sources of Distraction from a Cognitive Load Perspective. International J Scholarship Teaching Learning.

